# The Role of Artificial Intelligence in Cardiovascular Disease Risk Prediction: An Updated Review on Current Understanding and Future Research

**DOI:** 10.2174/011573403X351048250329170744

**Published:** 2025-04-17

**Authors:** Angad Tiwari, Purva C. Shah, Harendra Kumar, Tanvi Borse, Anjali Raj Arun, Manognya Chekragari, Sidhant Ochani, Yash R. Shah, Adithan Ganesh, Rezwan Ahmed, Ashish Sharma, Maneeth Mylavarapu

**Affiliations:** 1 Department of Internal Medicine, Maharani Laxmi Bai Medical College, Jhansi, Uttar Pradesh, India;; 2 Department of Internal Medicine, Rochester General Hospital, Rochester, NY 14621, USA;; 3 Department of Internal Medicine, Dow University of Health Sciences, Karachi, Pakistan;; 4 Department of Internal Medicine, Parkview Health, Fort Wayne, ID 46845, USA;; 5 Department of Pediatrics, School of Medicine & Health Sciences, University of North Dakota, Grand Forks, ND 58203, USA;; 6 Department of Pediatrics, College of Medicine, University of Florida, Gainesville, FL 32610, USA;; 7 Department of Internal Medicine, Khairpur Medical College, Khairpur Mir's, Pakistan;; 8 Department of Internal Medicine, Trinity Health Oakland, Pontiac, MI 48341, USA;; 9 Department of Internal Medicine, Icahn School of Medicine, Mount Sinai Elmhurst Hospital Center, Queens, NY 11373, USA;; 10 Department of Internal Medicine, Texas A&M School of Medicine, Houston Methodist Hospital, Houston, TX 77030, USA;; 11 Department of Internal Medicine, University of Connecticut, Farmington, CT 06269, USA;; 12 Department of Public Health, Adelphi University, NY 11530, USA

**Keywords:** Cardiovascular disease, artificial intelligence, computational intelligence, computer reasoning, machine intelligence, adverse cardiac event

## Abstract

Cardiovascular disease (CVD) Continues to be the leading cause of mortality worldwide, underscoring the critical need for effective prevention and management strategies. The ability to predict cardiovascular risk accurately and cost-effectively is central to improving patient outcomes and reducing the global burden of CVD. While useful, traditional tools used for risk assessment are often limited in their scope and fail to adequately account for atypical presentations and complex patient profiles. These limitations highlight the necessity for more advanced approaches, particularly integrating artificial intelligence (AI) into cardiovascular risk prediction. Our review explores the transformative role of AI in enhancing the accuracy, efficiency, and accessibility of cardiovascular risk prediction models. The implementation of AI-driven risk assessment tools has shown promising results, not only in improving CVD mortality rates but also in enhancing quality of life (QOL) markers and reducing healthcare costs. Machine learning (ML) algorithms predicted 2-year survival rates after MI with improved accuracy compared to traditional models. Deep learning (DL) forecasted hypertension risk with a 91.7% accuracy based on electronic health records. Furthermore, AI-driven ECG (Electrocardiography) analysis has demonstrated high precision in identifying left ventricular systolic dysfunction, even with noisy single-lead data from wearable devices. These tools enable more personalized treatment strategies, foster greater patient engagement, and support informed decision-making by healthcare providers. Unfortunately, the widespread adoption of AI in CVD risk assessment remains a challenge, largely due to a lack of education and acceptance among healthcare professionals. To overcome these barriers, it is crucial to promote broader education on the benefits and applications of AI in cardiovascular risk prediction. By fostering a greater understanding and acceptance of these technologies, we can accelerate their integration into clinical practice, ultimately aiming to mitigate the global impact of CVD.

## 1 INTRODUCTION

Cardiovascular disease (CVD) contributes to 32% of deaths worldwide and has the highest mortality rate of all diseases [1]. Outside of fatalities, CVD severely impacts an individual’s quality of life (QOL) and has contributed to an increased rate of hospital admissions, accounting for 7.6% to 21% of national health expenditures, which is a significant financial burden on the public health sector [2-4]. Therefore, clinicians must use effective CVD risk prediction tools to aid in the management of this disease.

Risk prediction models enable the customization of healthcare and resource streamlining [2]. By improving risk comprehension and enabling shared decision-making, risk prediction improves patient compliance and outcomes [2-4]. However, traditional risk assessment tools based on the presence of comorbidities such as diabetes mellitus, hypertension, smoking and hyperlipidemia, though effective, could underestimate the risk in high-risk, atypical patients despite their accuracy. Given that 20% of these patients do not possess common risk factors and 40% exhibit only one, traditional tools do not provide a holistic representation of the total risk analysis. Furthermore, factors such as time constraints, clinician uncertainty, and dilemmas regarding tool selection have impeded the use of conventional tools [5, 6]. Adopting artificial intelligence (AI) would help alleviate these challenges.

The integration of AI, particularly machine learning (ML) and deep learning (DL), combined with big data mining techniques, has made it possible to analyze large and complex datasets in a fraction of the time with minimal human intervention [7, 8]. While ML enables algorithms to learn patterns from data, DL utilizes artificial neural networks to automatically extract intricate details from the given information. In the context of cardiovascular risk prediction, ML and DL enable the discovery of connections that extend beyond conventional risk determinants such as genetics, lifestyle, and environmental factors, thereby providing highly personalized and accurate risk profiles [9]. Through continuous analysis of patient data, AI algorithms can dynamically adjust risk predictions over time and constantly evolve with an individual’s health journey [10].

Current AI-based models have consistently exhibited superiority in various aspects of cardiovascular risk prediction, including risk stratification and prognostication [11-16]. By expanding CVD detection through diagnostic image analysis, AI frameworks can facilitate treatment planning and disease forecasting, such as improving predictions for hospitalization and mortality [17-19]. Thus, AI risk prediction algorithms may also play a pivotal role in strengthening current screening, monitoring, and overall public health guidelines [19-21].

While AI technology has made significant progress, its practical implementation in the healthcare sector remains limited [22, 23]. A notable gap exists in understanding the real-world implementation of AI across healthcare, with limited evidence evaluating its performance, scalability, and impact on decision-making. Ethical implications and real-time feasibility are also understudied. Additionally, the long-term benefits of integrating AI risk prediction models into clinical practice lack robust research. Although previous reviews have touched on aspects of AI in CVD risk prediction, a comprehensive analysis synthesizing the evidence on real-world implementation challenges, ethical considerations, and long-term impact on patient outcomes and healthcare systems is lacking. This review aims to fill this gap by providing a comprehensive and critical analysis of the current landscape, highlighting key challenges and opportunities for the successful integration of AI in CVD risk prediction.

## 2 REVIEW

### 2.1 Conventional Risk Prediction Models

Conventional cardiovascular disease (CVD) risk prediction models continue to be the gold standard when determining management plans [2]. As cardiovascular disease continues to be the leading cause of mortality worldwide, a number of these prediction models have been developed over time, for example, the pooled cohort equation, the QRISK3 score, and the Framingham risk score. However, the quest to find an all-inclusive risk prediction model continues. The Framingham Heart Study was one of the first studies that pioneered the concept of utilizing risk factors as a tool to predict the risk of a cardiac-related event. It started with its first cohort in 1948 and is currently an ongoing project, now with its third generation of participants. Following this landmark research project, various studies were undertaken that incorporated different types of risk factors and their predictive abilities. Conventional risk prediction models have been compared in Table **1**.

As seen from the table, even though conventional risk prediction models are widely used due to their ease of usage and standardized use, they come with their limitations. Most scores are limited due to the lack of external validity [5, 24-26]. Available CVD risk prediction models are built predominantly on data from individuals who were Caucasian and represented high-income country populations, which thereby limits its overall predictive value and translation to lower- and middle-income populations [27, 28]. Another issue is that many of the existing CVD prediction models use multivariable regression methods to build prediction models linearly, but generally exhibit modest predictive performance, especially for certain subpopulations.

Thus, the use of conventional risk predictors should be interpreted as a weather forecast for patients and clinicians to decide if the risk is high enough to consider carrying an umbrella (for instance, taking a statin to reduce cardiovascular risk). These risk estimates should act as the starting point for patient-clinician discussions and not be viewed as a precise decision-making tool that determines the need for a medication or the lack thereof [29].

### 2.2 Artificial Intelligence-based Risk Prediction Models

#### 2.2.1 Machine Learning (ML)

ML uses four types of learning methods: 1) supervised, 2) unsupervised, 3) semi-supervised, and 4) reinforcement learning [30, 31]. Supervised learning uses a set of training examples, also known as labelled data, to learn from previous datasets to predict the output value for the new data [31]. With unsupervised learning, the computer detects unlabelled data without prior categorization of the dataset [30]. This learning method can be used in descriptive modelling and the detection of patterns. Semi-supervised learning uses information from both labelled and unlabelled data and, thereby, falls between these two [30]. Reinforcement learning enables the software to learn and improve the predictive model by determining ideal behaviours within a specific context and providing feedback [30]. By using these learning techniques, AI plays a critical role in the advancement of risk prediction models for cardiovascular diseases. A few of the commonly used machine learning algorithms to predict cardiovascular diseases include Artificial Neural Network (ANN), Deep Neural Network, Decision Tree, Fuzzy Logic, K-Nearest Neighbor (KNN), Naïve Bayes, and Support Vector Machine (SVM) [32]. The objective of these algorithms is to manage a vast amount of data to build a risk prediction model that has an exceptional ability to detect the disease at the earliest stages by detecting potential risk factors. This thereby promotes early detection and prevention of the disease, contributing to reduced mortality and hospital readmission rates.

#### 2.2.2 Deep Learning (DL)

Deep learning, inspired by human brains, identifies patterns in data through artificial neural networks [33]. Armed with millions of artificial neurons, DL can interpret cardiac imaging with millions of artificial neurons, enabling unambiguous risk assessment and, thereby, prompt intervention [34]. DL in risk prediction models adds objectivity and consistency, which is difficult to achieve when solely using human interpretation. Recent studies have highlighted its accuracy in spotting heart disease from images and extracting insights from electrocardiogram (ECG) data, such as predicting left ventricular systolic dysfunction (LVSD) [16-19].

#### 2.2.3 Big Data Mining

Big data mining extracts valuable insights from extensive datasets containing patient parameters, genetics, biomarkers, and social determinants of health. This identifies population-wide trends, aiding targeted preventive measures [35]. Integrating big data mining with machine learning and deep learning strengthens cardiovascular risk prediction potential, facilitating precision medicine and proactive strategies for reducing CVD impact [36]. Combining big data with AI not only enhances accuracy but also discovers novel risk factors, bolstering model predictive power [37]. A few examples of studies that have been conducted by researchers to evaluate the performance of AI-based prediction models in assessing cardiovascular disease risk are shown in Table **2**.

Conventional risk prediction models have well-established and extensively studied risk factors such as age, gender, blood pressure, family history, and cholesterol levels, which make it easier for healthcare professionals to communicate a patient’s risk for CVD. AI-based models, on the other hand, use machine learning algorithms to analyse large sets of data obtained from patients' electronic medical records, imaging, genetic factors, and lifestyle information, which helps identify subtle patterns and associations with a higher degree of accuracy. Additionally, AI models can also be updated with real-time data to ensure that they are up to date with current medical advancements. A combined approach of AI and conventional models could provide more accurate, holistic, and efficient predictions and insights into improving patient care and clinical outcomes.

AI-based models have shown exceptional accuracy in detecting ventricular arrhythmias, achieving a sensitivity and specificity of 99.2-98.8%, surpassing traditional classifiers [38, 39]. Regarding predictive accuracy, AI also excels in CVD risk prediction by leveraging big data analytics, potentially improving upon the moderate c-statistics of traditional models like congestive HF, hypertension, age ≥75 years, diabetes mellitus, and prior stroke, vascular disease, age 65 to 74 years, and sex category (CHA2DS2-VASc) and Framingham risk score [40], allowing for more accurate and personalised risk assessments.

AI-driven ECG analysis has also demonstrated promising results in detecting AF with a sensitivity of 79% and specificity of 79.5% and left ventricular systolic dysfunction (LVSD) with a sensitivity of 86.3% and specificity of 85.7%, suggesting that AI can enhance the diagnostic capabilities of ECGs [41, 42].

### 2.3 Application of AI-based Prediction Models

An early estimate based on a risk prediction model has the potential to greatly improve the outcome and prognosis of a certain disease. Multiple indicators of the efficiency of AI are exhibited in Fig. (**1**). Prevention stands as the paramount and most efficacious approach to mitigating the impact of CVD [43, 44]. Risk prediction is at the cornerstone of formulating treatment strategies, with contemporary guidelines advocating for cardiovascular risk-reducing strategies.

#### 2.3.1 Real-time Prognostication of MI Risk in Hospitalized Patients

AI-based models can offer real-time prognostic capabilities that hold significant value in time-critical situations, particularly during emergencies. For example, in a study involving 234,815 patients evaluating the efficiency of an AI-based early warning risk prediction model for the occurrence of in-hospital myocardial infarction (MI), the application of an MI risk prediction model enabled real-time monitoring in hospitalized patients, leading to automatic early warnings. This supported the consequent establishment of regional MI prevention and management systems, improved non-cardiovascular physicians' awareness of MI risk, and provided reference data for cardiovascular physicians. Additionally, the model offered decision-making support to primary care physicians, aiding in enhancing the understanding of disease characteristics, formulating effective treatment plans, optimizing medical resource allocation, and promoting hierarchical diagnosis and treatment strategies [45-47].

In another study, Wallert *et al*. [46] used four prevalent machine learning algorithms to analyze a dataset consisting of 51,943 instances of new-onset MI, which resulted in the creation of a robust prediction model for forecasting the 2-year survival rate following the initial onset of MI.

#### 2.3.2 Prognostication of Tachyarrhythmia Risk Post-MI

The presence of arrhythmia in conjunction with acute myocardial infarction (AMI) stands as a significant catalyst for the deterioration of cardiac function and heightened mortality risk [48, 49]. Substantiated research underscores that amongst patients undergoing percutaneous coronary intervention (PCI), the occurrence of arrhythmia before and after cardiac catheterization aligns with increased mortality risks [50]. Consequently, elucidating the risk determinants for post-MI arrhythmia and prognosticating its manifestation in afflicted individuals can heighten medical vigilance and enhance patient prognosis, Wang *et al*. [49] demonstrated machine learning algorithms consisting of 15 clinical variables executed to forge a robust framework for post-MI tachyarrhythmias. This innovative strategy exhibited pronounced superiority over the conventional Global Registry of Acute Coronary Events (GRACE) model. Timely anticipation of tachyarrhythmias during the acute phase of MI holds pivotal significance in informing clinical judgments. This investigation highlights the instrumental role of machine learning techniques in refining risk evaluation and advancing precision in clinical decision-making. AI models’ ability to detect early signs of deterioration and predict adverse events enables proactive measures like closer monitoring or prophylactic medication to prevent life-threatening complications. These advancements highlight the potential of AI to not only predict risk but also to guide timely interventions, potentially leading to improved patient outcomes and reduced healthcare costs.

#### 2.3.3 Prognostication of Hypertension Risk based on Gene Expression

AI is being leveraged to enhance our understanding of the intricate factors contributing to the development and physiological processes underlying hypertension (HTN). AI stands as a pivotal instrument in crafting tailored therapeutic strategies for individuals affected by HTN. Held *et al*. [50] exhibited the superiority of ML over a logistic regression model in the realm of prognosticating HTN using gene expression data. Another study by Li *et al*. [51] demonstrated that ML, particularly employing the support vector machine algorithm, effectively identified a total of 177 novel genes with potential associations with HTN. Subsequent investigations have further elucidated that the judicious application of machine learning has demonstrated promising results and commendable proficiency in meticulously prognosticating the susceptibility to hypertension through the amalgamation of environmental and genetic determinants [52].

#### 2.3.4 Prognostication of Hypertension Risk based on Electronic Patient Data

An investigation by Maxwell *et al*. [53] involving 110,300 subjects revealed the predictive prowess of deep learning (DL) in the realm of hypertension prognosis. The model successfully anticipated hypertension risk through an intricate fusion and analysis of four physiologic parameters, twenty-six haematological markers, twelve urinalysis results, and a composite score of parameters extracted from hepatic functional assessments. Furthermore, the insights provided by Ye *et al*. [54] highlight the proficiency of machine learning, particularly when employing K-nearest neighbour algorithms in scrutinizing inputs culled from electronic health records (EHR) to predict hypertension risk. In this retrospective cohort comprising 823,627 subjects, machine learning showcased an impressive predictive accuracy of 91.7%, while the validation cohort, comprised of 680,810 subjects, exhibited a commendable predictive accuracy of 87% in a prospective context.

#### 2.3.5 Warfarin Dosing based on Pharmacogenomics

AI has also carved its niche in the domain of cardiovascular drug therapy. The synergy of AI applications, big data and precision medicine has revolutionized drug development, facilitating the discovery of effective treatment modalities with a reduced risk of side effects [55]. The integration of pharmacogenomics and precision medicine has had a significant influence on warfarin dosing in diverse patient populations, as demonstrated in the randomized clinical trials conducted by Pirmohamed *et al*. [56]. In line with research findings, it was demonstrated that patients on pharmacogenetic-based warfarin dosing achieved a significantly higher time within the therapeutic International Normalized Ratio (INR) range compared to those on standard dosing. Similar favourable outcomes were observed in the Asian patient population undergoing warfarin therapy, as reported by Syn *et al*. [57].

#### 2.3.6 Screening for Left Ventricular Systolic Dysfunction using Wearable Technology

Individuals with left ventricular systolic dysfunction (LVSD) face a significantly increased risk of heart failure, over eight times more compared to those without it. Moreover, their likelihood of experiencing premature mortality is almost twice as high as individuals without LVSD [58]. AI-assisted electrocardiography (AI-ECG) has emerged as a reliable screening modality for the identification of LVSD, substantiated by its algorithmic architecture honed through the analysis of 12-lead ECGs procured under clinical settings [59]. Enhanced inclusivity facilitated by wearable and portable technologies has the potential to extend the reach of AI-driven screening methods. Analysis carried out by Khunte *et al*. [59] has been adeptly tailored to accommodate the intricacies associated with single-lead ECGs captured by wearable and portable devices, where noise can significantly affect the results and interpretation. Under this blueprint, a noise-adapted deep learning algorithm, which exhibits remarkable precision in the identification of LVSD using single-lead ECG data, has been devised. Notably, the algorithm’s resilience to significant noisy artifacts is striking, despite these specific disturbances not being encountered during its developmental phase. In addition, the algorithm demonstrated effectiveness even when exposed to ECGs infused with a noise-to-signal ratio that was double that of the essential physiological signals. These attributes are promising for the implementation of screening strategies reliant on everyday wearable device platforms.

### 2.4 Limitations, Challenges, and Critical Appraisal of AI-based Prediction Models

AI-based prediction models have sparked significant interest and promise in revolutionizing a variety of disciplines of healthcare, including CVD risk assessment tools. However, these models have inherent limitations and hurdles that need thorough investigation by cardiovascular health specialists and researchers to be implemented into clinical practice. It is imperative for clinicians to cautiously engage with these models while analyzing their validity and the presence of any biases. Ethical guidelines and data protection acts must also be strictly followed, and efforts to develop and incorporate AI into medical care must be both rigorous and collaborative. Data bias is a potential limitation in revolutionizing a variety of disciplines of healthcare and can be addressed by actively seeking and incorporating datasets that represent diverse demographics, such as race, ethnicity, gender, socioeconomic status, and geographic location. Furthermore, data sharing should be incentivized across institutions to promote the creation of large, more diverse datasets. In regards to ethical concerns, developing and implementing comprehensive AI ethics guidelines addressing issues like patient autonomy, data privacy, algorithmic transparency, and accountability could help eliminate these ethical concerns.

As the use of AI in healthcare grows, addressing these challenges is critical to realizing and utilizing the full promise of AI-based prediction models while Maintaining the highest standard of patient care. Furthermore, the scalability of AI-based solutions in low-resource settings remains a concern due to the potential demands for computational power and complex algorithms.

The challenges and current disadvantages of AI-prediction models can be further divided into:

#### 2.4.1 Reliance on Data Accuracy

One of the major disadvantages of AI-based prediction models is their reliance on high-quality and comprehensive data [60]. To make trustworthy predictions, these models need massive datasets spanning various patient groups, medical histories, and risk indicators. However, data quality, incompleteness, and other biases may jeopardize the reliability of these models, resulting in inaccurate conclusions and treatment suggestions. Furthermore, the demand for huge amounts of computer power and complex algorithms may present logistical challenges, particularly for smaller healthcare facilities with limited resources [60, 61].

Cardiovascular health professionals must also extensively assess the accuracy and generalizability of AI-based predictions. They must examine the basic assumptions, methods, and algorithms that underpin these models [61]. Transparency and interpretability are critical, as is understanding how AI makes predictions. The ‘black box’ nature of some AI systems raises concerns about their ability to convey predictions to patients and colleagues, thereby limiting their confidence and adoption into clinical care [61, 62]. Moreover, clinicians should inquire about the underlying data sources and demographics used to train the AI model, as well as examine its potential biases and generalizability across different patient groups. Understanding the algorithmic method and openness in decision-making is critical for developing confidence and interpretability, especially when communicating this risk to patients [63]. Doctors should also investigate the model's performance metrics and validation procedures to determine its accuracy and reliability in real-world clinical settings. Addressing these challenges allows for a thorough evaluation of AI-based models, allowing for informed and responsible adoption into the treatment of cardiovascular patients.

#### 2.4.2 Patient Data Safety

Ethical concerns must also be addressed when incorporating AI-based models into health care. Protecting patient privacy and data security is critical, especially given the sensitive nature of personal health information [26, 64]. The possibility of bias, either due to data sources or algorithmic design, demands ongoing monitoring to prevent worsening health disparities. Furthermore, the use of AI in medical decision-making poses issues of responsibility and liability when errors occur. To ensure patient safety and professional standards, it is critical to strike a balance between AI’s capabilities and human supervision [65].

#### 2.4.3 Need for Technical Prowess in Clinicians

To effectively translate AI-generated insights into appropriate treatment recommendations, cardiovascular health professionals must bridge the gap between data scientists and physicians. This requires specialized training and ongoing collaboration to ensure proper model deployment and interpretation. Furthermore, incorporating AI into existing healthcare procedures and electronic health record systems may be difficult and time-consuming, particularly in non-urban hospitals, reducing clinical efficiency [63, 65].

Cardiovascular health clinicians must also cautiously engage with these models, searching for validity and any biases. Ethical guidelines and data protection acts must be strictly followed, and efforts to comprehend and incorporate AI in medical care must be rigorous and collaborative. Table **3** delineates a comprehensive array of appraisal aspects crucial for gauging the quality and validity of AI-driven medical prediction models. Derived from the work of van Smeden *et al*., this framework encompasses various dimensions, including the necessity of AI integration, data representativeness, model evaluation comprehensiveness, and adherence to reporting guidelines, offering a structured approach to meticulous evaluation [20].

### 2.5 Recommendations

Cardiovascular research has witnessed significant advancements in recent years, with AI holding potential to revolutionize the diagnosis and management of cardiovascular diseases [63]. AI algorithms can process large amounts of patient data, including medical records, lab results, and imaging studies, to identify patterns and risk factors that may not be immediately apparent to healthcare providers when using traditional risk prediction models [63]. These algorithms can be trained to predict the likelihood of an individual developing cardiovascular disease and can assist in determining the most appropriate treatment plan for each patient.

By leveraging the power of AI, healthcare providers can potentially intervene earlier in disease progression, improving outcomes and reducing the burden on the healthcare system. Potential future innovations can be categorized based on their feasibility in the short-term *versus* long-term [64-67]:

Short term: 1) AI-driven personalized treatment plans that can analyze information on genetics, lifestyle, and patient-specific medical history to identify potential risks and treatments, 2) continuous and remote monitoring using wearable devices collecting real-time data on various metrics, 3) patient education platforms for providing personalized information and recommendations to promote healthier lifestyles and treatment adherent plans.

Long term: 1) early detection of disease through image analysis of echocardiograms, MRIs, and CT scans, 2) predictive analytics for disease risk assessment, analyzing a combination of factors including biomarkers, patient data, and lifestyle variabilities, 3) AI-assisted drug discovery by simulating molecular interaction and predicting the effectiveness of new compounds, 4) actions to emergent events and optimization of treatment strategies such as medication dosages through real-time data analysis and feedback, 5) genomic analysis to identify genetic predispositions for preventive measures, 6) data integration and fusion of patient profiles such as electronic health records, wearable devices, and genetic information all in one place to provide a comprehensive view of individuals’ CV health, 7) AI simulations to model disease progression under various CV system states to design intervention and predict outcomes, and lastly 8) clinical trial optimization to help design, identify suitable patients, predict treatment response and monitor progress for the trials, which may streamline the drug development process and reduce costs. These advancements in AI have the potential to revolutionize cardiovascular care by enabling precise diagnoses, tailored interventions, and, ultimately, improving patient outcomes. Selecting proper algorithms, reporting all evaluation matrices, comparing them to current standards, and validating cohorts are recommended for the correct clinical context and interpretation [64, 65]. As healthcare workers and policymakers, caution should be taken when considering variability when using AI models for decision-making.

Furthermore, clinician education should prioritize foundational knowledge of AI concepts, hands-on training with AI-based tools, ethical considerations, and continuous learning opportunities. This multifaceted approach will empower clinicians to interpret and utilize AI-generated insights in their clinical decision-making effectively. Additionally, integrating AI into healthcare systems requires robust data infrastructure, user-friendly interfaces, and integration with clinical decision support systems. Successful implementation also requires ensuring scalability and accessibility for diverse healthcare settings, as well as continuous evaluation and monitoring of AI performance.

## CONCLUSION

The integration of AI into cardiovascular disease risk prediction can potentially transform patient care by enhancing early detection, personalized treatment plans, and disease management strategies. Although AI-based models provide a comprehensive and dynamic assessment compared to the existing traditional methods- they also present challenges related to the accuracy of data, transparency, and ethical considerations. As the field of AI in medicine and healthcare continues to evolve, clinicians must approach AI tools with caution, ensuring that these technologies will complement human expertise well while maintaining patient safety and addressing potential biases.

To fully realize the potential of AI in cardiovascular care, future research should prioritize several key areas. Rigorous real-world validation of AI models is crucial to confirm their performance and generalizability across diverse populations and clinical settings. Furthermore, developing equity-focused AI models that address potential biases and ensure equitable outcomes for all patient populations is essential to avoid exacerbating existing health disparities. Research on explainable AI (XAI) can enhance transparency and trust in AI-based predictions, enabling clinicians to better understand and communicate risk assessments to patients. Additionally, studies evaluating the seamless integration of AI tools into existing clinical workflows are needed to optimize efficiency and minimize disruption. Finally, assessing the cost-effectiveness of AI-based solutions is crucial for informing healthcare policy and resource allocation decisions. By addressing these research priorities, we can pave the way for the responsible and effective implementation of AI in cardiovascular risk prediction, ultimately improving patient care and outcomes.

## Figures and Tables

**Fig. (1):**
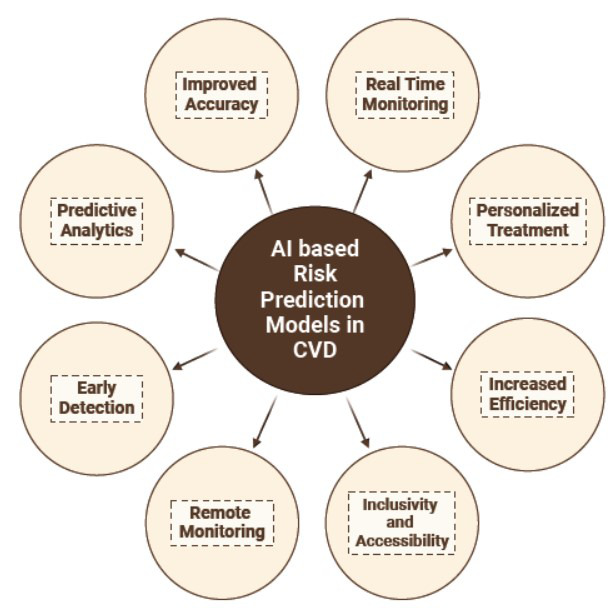
Advantages of AI-based risk prediction models in cardiovascular disease (CVD). **Note:** Key advantages of AI-based risk prediction models in Cardiovascular Disease (CVD). These models enhance real-time monitoring, improve accuracy, enable remote monitoring, promote inclusivity and accessibility, increase efficiency, provide personalized treatment, facilitate early detection, and leverage predictive analytics for better patient outcomes.

**Table 1:** A stipulated comparison of various cardiovascular risk prediction models.

**S. No.**	**Risk Predictor**	**Criteria**	**Outcome**	**Challenges**
1	Pooled cohort equation (PCE)	Age, sex, SBP, treatment for hypertension, TC, HDL-C, history of T2DM and smoking status	10-year risk of a nonfatal MI, CHD death and fatal or nonfatal stroke	Does not involve ‘novel’ risk markers, some concerns about potential over-estimation of risks [3, 4].
2	Framingham risk score	Age, sex, SBP, TC, T2DM and smoking	10-year risk of a nonfatal MI and CHD death	Accuracy is somewhat limited in applicability amongst certain specific populations as it does not consider ethnicity, family history or socio-economic factors. May overestimate risk in countries with a low CVD mortality rate and *vice versa* [5].
3	QRISK3 score	Age, sex, SBP, TC/HDL-C ratio, T2DM, smoking status, ethnicity, social deprivation, body mass index, family history of CHD in a first-degree relative younger than 60 years, treated hypertension, rheumatoid arthritis, atrial fibrillation, stage 4 or 5 chronic kidney disease, migraine, corticosteroid use, systemic lupus erythematosus, treatment with atypical antipsychotic medications, severe mental illness, erectile dysfunction, and variability of blood	10-year risk of cardiovascular events	Based on routinely collected data from patients’ electronic health records (EHRs). QRISK does not fully capture the variability between patients and medical practices, which leads to uncertainty in predicting individual CVD risk. There is significant variability within each practice in recording EHRs due to differences in computer systems and clinical coding. Quality of data from different practices can vary, and this affects patients’ QRISK scores [6, 7].
4	Systematic coronary risk evaluation	Age, sex, SBP, TC and smoking status	10-year risk of cardiovascular mortality	Only predicts risk of CV death - does not consider nonfatal CVD events. May overestimate risk in countries with a decreasing CVD mortality and underestimate risk in countries with increasing CVD mortality. May underestimate risk in patients with diabetes mellitus, central obesity, family history of premature CVD, low HDL, or elevated triglyceride, fibrinogen, lipoprotein A and B, high-sensitivity CRP, or homocysteine levels [8, 9].
5	Assign risk score	Age, sex, SBP, TC, T2DM, smoking, social deprivation, and family history of CVD	10-year risk of cardiovascular events	Based on data limited to Scottish populations. Does not consider obesity or BMI [9, 10].
6	CUORE risk score	Age, sex, SBP, TC, HDL-C, presence of T2DM, treatment for hypertension and smoking status	10-year risk of CHD and cerebrovascular events	Not applicable in patients > 65 years. Based on data limited to Italian populations [11].
7	Reynolds risk score	Age, sex, SBP, TC, HDL-C, HbA_1c_ if diabetic, smoking, hsCRP and parental history of MI before the age of 60 years	10-year risk of cardiovascular events	Limitations related to ethnicity, socioeconomic status, and age as the score was developed based on data from Caucasian patients aged 45 years or older who live in developed countries [7].
8	Prospective cardiovascular munster risk score	Age, SBP, LDL-C, HDL-C, triglycerides, presence of T2DM, family history of MI and smoking status	10-year risk of fatal or nonfatal CHD event	Only based on data from German males. Only estimates risk for fatal or nonfatal myocardial infarction and acute coronary death [8].

**Abbreviations:** SBP= Systolic Blood Pressure, TC= Total Cholesterol, HDL-C= High Density Lipoprotein-Cholesterol, LDL-C= Low Density Lipoprotein-Cholesterol, T2DM=Type 2 Diabetes Mellitus, MI= Myocardial Infarction, CHD= Coronary Heart Disease, CVD=Cardiovascular Disease, hsCRP= high sensitivity C-reactive Protein, BMI= Body Mass Index.

**Table 2:** Studies exploring the role of artificial intelligence in cardiovascular medicine.

**Authors**	**Year of Study**	**Study Design**	**Type of Study**	**Type(s) of Machine Learning Technique**	**Data Size**	**Performance**
Moghaddasi *et al.* [34]	2016	Prospective	Assessment of Mitral regurgitation using echocardiography images	SVM, linear discriminant analysis, template matching	102 patients were categorized as follows: mild MR (n=34), moderate MR (n=32) and severe MR (n=36)	Sensitivity: 99.38% Specificity: 99.63%
Khamis *et al.* [35]	2017	Retrospective	Automatic apical view classification of echocardiograms	Multistage classification and supervised learning	309 clinical echocardiogram clips of apical views	Accuracy: A2C: 97% A4C: 91% ALX: 97%
Sengupta *et al.* [36]	2017	Retrospective	Differentiation of restrictive cardiomyopathy and constrictive pericarditis by machine learning	Associative memory classifier / Machine learning	Clinical and echocardiographic data of patients as follows: constrictive pericarditis (n=50), restrictive cardiomyopathy (n=44)	AUC: 96.2%
Ala *et al.* [37]	2019	Prospective	Cardiovascular risk prediction using ML techniques in patients with no history of cardiovascular disease at baseline.	Auto Prognosis *via* SVM, RF, KNN	423,604 participants without cardiovascular disease at baseline	AUC-ROC: 0.774, 95% CI: 0.768-0.780
Zreik *et al.* [38]	2018	Retrospective	Automatic identification of functionally significant coronary artery stenoses in patients	CNN, Computer-Aided Engineering (CAE), and SVM.	Coronary CT Angiography scan of 166 patients who underwent invasive Fractional Flow Reserve (FFR) measurements	Sensitivity: 60%, 70%, 80% Specificity: 77%, 71%, 59% respectively
Attia *et al.* [39]	2019	Retrospective	Role of AI-based learning algorithms to diagnose asymptomatic left ventricular dysfunction.	CNN	52,870 patients with no symptoms of left ventricular dysfunction.	AUC: 93% Sensitivity: 86.3% Specificity: 85.7% Accuracy: 85.7%
Gessert *et al.* [40]	2019	Retrospective	Automatic plaque detection in coronary heart disease	CNN	Data set obtained from 49 *in vivo* images of patients	Sensitivity: 90.9% Specificity: 92.4% Accuracy: 91.7%
Kakadiaris *et al.* [41]	2018	Retrospective	Machine learning (ML)-based risk calculator for cardiovascular risk prediction	SVM	13-year follow up dataset from 6,459 participants who were free of cardiovascular disease at baseline	Sensitivity: 86% Specificity: 95% AUC: 92%

**Abbreviations:** AUC: Area Under the Curve; A2C: Apical Two-chamber; A4C: Apical Four-chamber; ALX: Apical Long Axis; SVM: Support Vector Machine; CNN: Convolutional Neural Network; KNN: K Nearest Neighbour, RF: Random Forest.

**Table 3:** Framework for critically appraising artificial intelligence-based prediction models in cardiovascular disease.

**Question**	**Appraisal Aspect**	**Description**
1	Necessity of AI	Assess whether the use of artificial intelligence is essential to address the specific medical issue at hand. Determine if conventional methods suffice.
2	Integration with clinical workflow	Evaluate how seamlessly the AI prediction model can be incorporated into the existing clinical practices without disrupting the established workflow.
3	Representativeness of data	Examine if the data used for developing and testing the prediction model accurately reflect the characteristics of the intended patient population.
4	Alignment of prediction timepoint	Verify if the timing of predictions aligns appropriately with the measurements of relevant features, ensuring clinical relevance and practicality.
5	Reliability of outcome labelling	Scrutinize the method used to label outcome variables, ensuring consistency, reproducibility, and independence in the process.
6	Adequacy of sample size	Determine whether the sample size employed for the AI model's development and testing is sufficient to yield reliable and robust predictions.
7	Mitigation of predictive optimism	Assess whether steps were taken to address the overestimation of predictive performance, providing a realistic representation of the model's capabilities.
8	Comprehensive model evaluation	Investigate if the assessment of the AI model's performance goes beyond basic classification statistics, encompassing a broader and meaningful evaluation.
9	Adherence to reporting guidelines	Verify whether the study adheres to established reporting guidelines for AI prediction models, ensuring transparency, reproducibility, and clarity.
10	Consideration of algorithmic fairness	Examines whether the model accounts for and addresses potential biases or unfairness in its predictions, promoting equitable and unbiased outcomes.
11	Sustainability and usability	Evaluate whether the developed AI prediction model is designed to be open for future testing, updates, and practical implementation in clinical settings.
12	Avoidance of overinterpretation	Ensures that the relationships between individual features and the outcome are presented accurately, avoiding unwarranted or exaggerated interpretations.

## LIST OF ABBREVIATIONS

AI: Artificial Intelligence
CVD: Cardiovascular Disease
DL: Deep Learning
ML: Machine Learning
QOL: Quality of Life
